# Endogeneity in Logistic Regression Models

**DOI:** 10.3201/eid1103.050462

**Published:** 2005-03

**Authors:** George Avery

**Affiliations:** * University of Minnesota, Duluth, Minnesota, USA

**Keywords:** letter, logistic regression, endogeneity, collinearity, hemolytic uremic syndrome, model specification

**To the Editor:** Ethelberg et al. (1) report on a study of the determinants of hemolytic uremic syndrome resulting from Shiga toxin–producing *Escherichia coli*. The dataset is relatively small, and the authors use stepwise logistic regression models to detect small differences. This indicates that the authors were aware of the limitations of the statistical power of the study. Despite this, the study has an analytical flaw that seriously reduces the statistical power of the study.

An often overlooked problem in building statistical models is that of endogeneity, a term arising from econometric analysis, in which the value of one independent variable is dependent on the value of other predictor variables. Because of this endogeneity, significant correlation can exist between the unobserved factors contributing to both the endogenous independent variable and the dependent variable, which results in biased estimators (incorrect regression coefficients) (2). Additionally, the correlation between the dependent variables can create significant multicollinearity, which violates the assumptions of standard regression models and results in inefficient estimators. This problem is shown by model-generated coefficient standard errors that are larger than true standard errors, which biases the interpretation towards the null hypothesis and increases the likelihood of a type II error. As a result, the power of the test of significance for an independent variable X_1_ is reduced by a factor of (1-r^2^_(1|2,3,….)_), where r_(1|2,3,….)_ is defined as the multiple correlation coefficient for the model X_1_ = f(X_2_,X_3_,…), and all X_i_ are independent variables in the larger model (3,4).

The results of this study clearly show that the presence of bloody diarrhea is an endogenous variable in the model showing predictors of hemolytic uremic syndrome, in that the diarrhea is shown to be predicted by, and therefore strongly correlated with, several other variables used to predict hemolytic uremic syndrome. Similarly, Shiga toxin 1 and 2 (*stx1, stx2)* genes are expected to be key predictors of the presence of bloody diarrhea, independent of strain, due to the known biochemical effects of that toxin (5,6). Because the strain is in part determined by the presence of these toxins, including both strain and genotype in the model means that the standard errors for variables for the Shiga-containing strains and bloody diarrhea symptom are likely to be too high, and hence the significance levels (p values) obtained from the regression models are higher than the true probability because of a type I error.

This flaw is a particular problem with studies that use a conditional stepwise technique for including or excluding variables. The authors note that they excluded variables from the final model if the significance in initial models for those variables was less than an α level (p value) of 0.05. Given the inefficiencies due to the endogeneity of bloody diarrhea, as well as those that may result from other collinearities significant predictors were likely excluded from the study, although this cannot be confirmed from the data presented.

The problems associated with the endogeneity of bloody diarrhea can be overcome by a number of approaches. For example, the simultaneous equations approach, such as that outlined by Greene (7), would have used predicted values of bloody diarrhea from the first stage of the model as instrumental variables for the actual value in the model for hemolytic uremic syndrome. Structural equations approaches, such as those suggested by Greenland (8), would also be appropriate. However, bloody diarrhea is not the only endogenous variable in their models, and extensive modeling would be necessary to isolate the independent effects of the various predictor variables. Given the small sample size, this may not be possible.

The underlying problem in the study is the theoretical specifications for the model, in which genotypes, strains, and symptoms are mixed, despite reasonable expectations that differences in 1 level may predict differences in another. For example, the authors’ data demonstrate that all O157 strains contain the *stx_2_* gene and have higher rates of causing hemolytic uremic syndrome and bloody diarrhea. This calls into question the decision to build an analytic model combining 3 distinct levels of analysis. Such a model depends on the independence of the variables to gain unbiased, efficient estimators. The model of the relationships one would develop from a theoretical perspective would predict the opposite (Figure). We expect that the genotypes (by definition) will predict the strain, and that strains have a differential effect on symptoms. The high level of intervariable correlation due to these relationships, coupled with the decision to exclude variables based on likely inefficient p values, raises questions concerning the reliability of the results and conclusions. In particular, the conclusions that strains O157 and O111 are not predictors of hemolytic uremic syndrome deserve to be revisited; other excluded variables may also be significant predictors when considered under an appropriate model. These problems point to the need to ensure proper specification of analytic models and to demonstrate due regard for the underlying assumptions of statistical models used.

**Figure:**
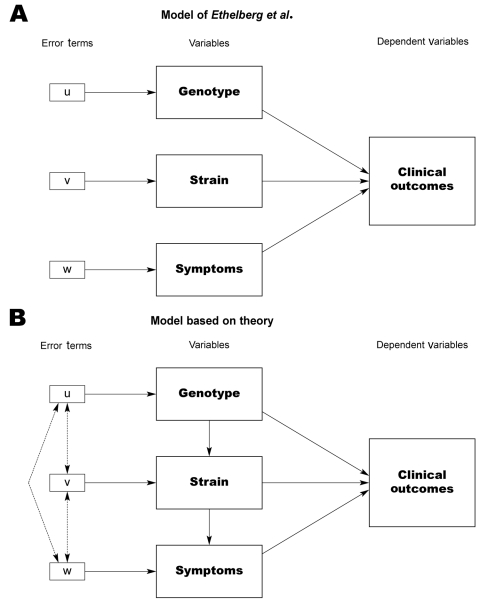
Model for determining virulence factors for hemolytic uremic syndrome.
