# Supplementary material for: Endogeneity in Logistic Regression Models
Source: Emerg Infect Dis. 2005 Mar;11(3):503–5. doi: 10.3201/eid1103.050462 (PMC3298240; doi:10.3201/eid1103.050462)
Supplement: Related texts

# Reply: Endogeneity in Logistic Regression Models

**DOI:** 10.3201/eid1103.050071

**Authors:** Steen Ethelberg, Kåre Mølbak

**Affiliations:** * Statens Serum Institut, Copenhagen, Denmark

**In response:** We appreciate Avery's interest (1) in our article (2), although we believe the critique of the methods is largely based on misunderstandings. We developed a model for the risk of progression to hemolytic uremic syndrome (HUS) containing 3 variables: whether the infecting Shiga toxin–producing *Escherichia coli* isolate had the *stx*_2_ gene, age of the patient, and occurrence of bloody diarrhea. The critique relates to the fact that bloody diarrhea and *stx*_2_ are not independent, since we showed that *stx*_2_ was strongly associated with progression to HUS (odds ratio [OR] = 18.9) and also weakly associated with development of bloody diarrhea (OR = 2.5) (2). Avery uses the term endogeneity as it is used in econometric analyses; however, the term "intermediary variable," i.e., a factor in the causal pathway leading from exposure to disease, is more frequently used in epidemiology. In this context, we chose to consider bloody diarrhea as a potential confounder (3). A confounder is a risk factor but is also independently associated with the exposure variable of interest and is not regarded as part of the causal pathway (Figure). Bloody diarrhea may act as a confounder if patients with bloody stools are treated differently by the examining physicians or if, for instance, unknown virulence factors contribute to the risk of having bloody stools.

**Figure:** A) Representation of the original model (2). B) Representation of the model with bloody diarrhea as an intermediary variable, which should not be adjusted for.

A second line of critique of our methods apparently develops from the idea that virulence factors determine the serogroup. This idea, however, is a biological misconception. In fact, virulence genes and serogroup are independent at the genetic level, and an important point of our article is that HUS is determined by the virulence gene composition of the strain rather than the serogroup.

Regardless of the status of the bloody diarrhea variable, excluding it from the model doesn't change the conclusions of the article. A revised model contains only the significant variables age and *stx*_2_ (Table). Serotype O157 is still not an independent predictor of HUS, and this result is robust.

**Table:** Risk factors for HUS among 343 STEC patients, Denmark 1997–2003, comparison of models with and without bloody diarrhea as a variable*

Determinant	No. of patients	No. (%) with HUS	Original model, OR (95% CI)	New model, OR (95% CI)
*eae*				
Negative	111	0 (0.0)		
Positive	232	21 (9.1)	NI	NI
*stx*_2_				
Negative	159	1 (0.6)	1	1
Positive	184	20 (10.9)	18.9 (2.4–146)	24.6 (3.2–187)
Age				
>8 y	178	3 (1.7)	1	1
<7 y	165	18 (10.9)	11.4 (3.2–41.3)	9.7 (2.7–34.1)
Bloody diarrhea				
No	218	6 (2.8)		
Yes	125	15 (12.0)	4.5 (1.6–12.7)	EX
O157				
No	262	10 (3.8)		
Yes	81	11 (13.6)	NS	NS

*HUS, hemolytic uremic syndrome; STEC, Shiga toxin–producing *Escherichia coli*; OR, odds ratio; CI, confidence interval; NI, not included (test not appropriate); NS, not significant; EX, excluded from model.

## References

[1] Avery G. Endogeneity in logistic regression models. Emerg Infect Dis. 2005;11:503-504.1578949210.3201/eid1103.050462PMC3298240

[2] Ethelberg S, Olsen KE, Scheutz F, Jensen C, Schiellerup P, Enberg J, Virulence factors for hemolytic uremic syndrome, Denmark. Emerg Infect Dis. 2004;10:842–7.1520081710.3201/eid1005.030576PMC3323205

[3] Griffin PM, Mead PS, Sivapalasingam S. *Escherichia coli* O157:H7 and other enterohaemorrhagic *E. coli*. In: Blaser MJ, Smith PD, Ravdin JI, Greenberg HB, Guerrant RL, editors. Infections of the gastrointestinal tract. Philadelphia: Lippincott Williams & Wilkins; 2002. p. 627–42.
